# Nutritional Rickets Presenting as Chronic Episodic Extremity Pain in a 9-year-old with Autism

**DOI:** 10.5811/cpcem.2018.2.37206

**Published:** 2018-07-16

**Authors:** Noopur Tripathi, Roopa Kanakatti Shankar, Aline Baghdassarian

**Affiliations:** * Virginia Commonwealth University School of Medicine, Richmond, Virginia; † Children’s Hospital of Richmond at VCU, Department of Pediatrics, Division of Pediatric Endocrinology, Richmond, Virginia; ‡ Virginia Commonwealth University School of Medicine, Department of Emergency Medicine, Division of Pediatric Emergency Medicine, Richmond, Virginia

## Abstract

Rickets due to vitamin D deficiency, typically presenting as bowed legs in toddlers, is uncommon in the modern era. We describe the case of a nine-year-old girl with autism and developmental delay who was evaluated for chronic intermittent extremity pain for more than one year prior to referral to the emergency department for hypocalcemia and increased alkaline phosphatase, which eventually led to the diagnosis of rickets confirmed by radiographic and laboratory findings. This report highlights the importance of the patient’s history of developmental delay and autism in the evaluation and approach to limb pain, and discusses the appropriate diagnostic approach.

## INTRODUCTION

Nutritional rickets is a metabolic bone disease marked by failure of bone mineralization and architectural disruption at the growth plate. Calcipenic rickets is due to a deficiency in calcium; it can occur either due to inadequate intake or metabolism of vitamin D, or inadequate intake or absorption of calcium in the setting of normal vitamin D levels. This commonly presents at an early age with delayed closure of the fontanelle, frontoparietal bossing of the skull, bowing of the legs, widening of the wrists, and craniotabes. Rickets can also cause developmental delay, delayed achievement of motor milestones, hypocalcemic seizures in the first year of life, and neurologic as well as cardiac pathology. While essentially eradicated in the developed world due to vitamin D supplementation of newborns, there are still reports of nutritional rickets worldwide. This case highlights the importance of considering rickets in the differential diagnosis, especially if there is history of developmental delay and autism.

## CASE REPORT

A nine-year-old Hispanic female with a past medical history of autism and global developmental delay presented to our emergency department (ED) complaining of a one-year history of pain in her extremities. The pain initially started in the right leg causing her to limp, trip, and fall. She was evaluated by her primary care physician and referred to a physical medicine and rehabilitation clinic that prescribed supramalleolar/ankle foot orthosis (SMAFO). The leg pain resolved, but she developed episodic pain in her bilateral upper extremities a month later. Initially manifesting as pain in her left arm, it was managed with nonsteroidal anti-inflammatory medications; then as this resolved she developed pain in her right arm. The episodic chronic pain in her extremities prompted laboratory evaluation and eventually her referral to the ED because of an elevated alkaline phosphatase (1,847 international units/liter [L]) and low serum calcium (6.4 milligrams [mg]/ deci-liter [dL]). Her past medical history was significant for autism and developmental delay. She did not have a family history of frequent fractures, bone pathology, or calcium problems.

On exam, she was non-verbal but followed commands and was comfortable with no acute distress. Her weight and height were less than the third percentile for age with minimal subcutaneous fat but normal body mass index (twelfth percentile). She had angular deformity and diffuse tenderness in the right and left arms and proximal forearms. She was able to bear weight but had lower extremity pain and difficulty with ambulation. The rest of her physical exam was normal. We noted no spine tenderness, brachydactyly or other dysmorphic features.

**Image 1:**
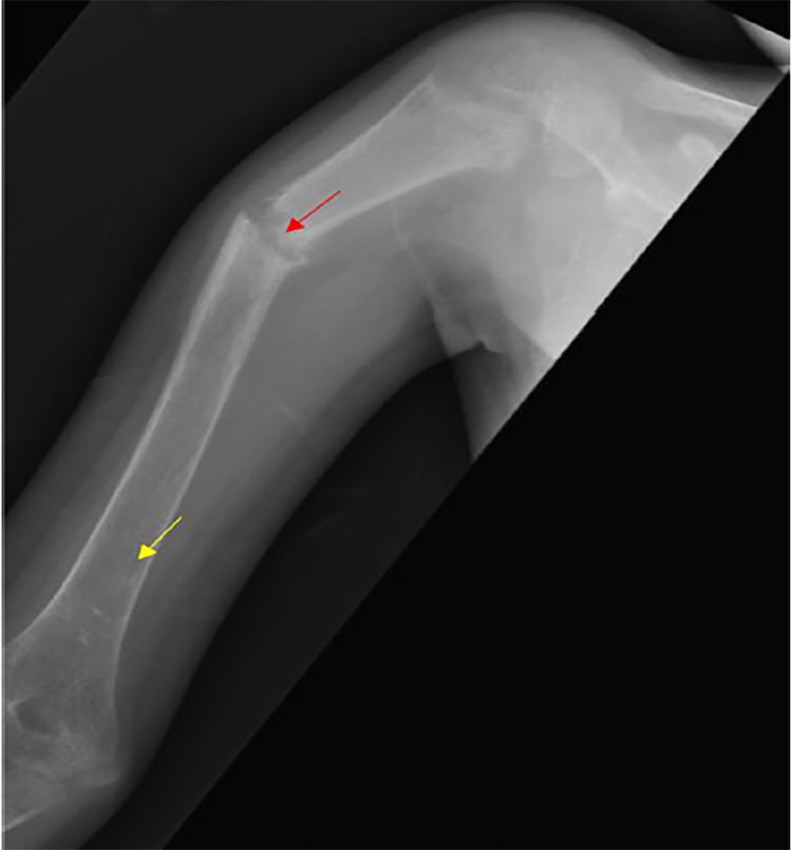
Radiograph of the right humerus of a nine-year-old female patient showing demineralization (yellow arrow) and healing fracture (red arrow).

**Image 2:**
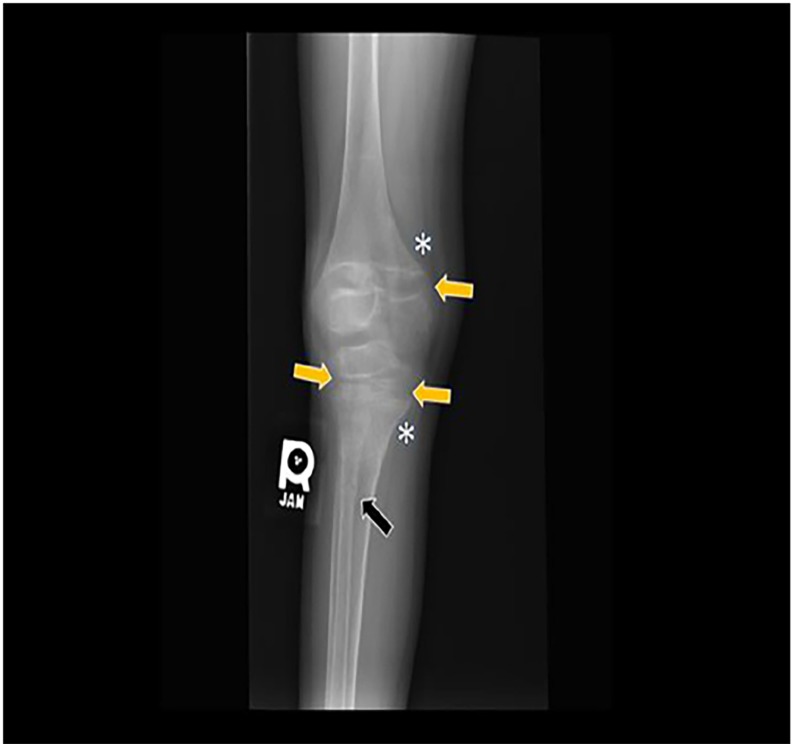
Marked generalized bony demineralization with widening of the physes in the distal femur, proximal tibia, and proximal fibula (yellow arrows) with metaphyseal fraying and flaring (asterisk). Healing nondisplaced transverse fracture at the proximal metadiaphysis of fibula (black arrow).

Initial laboratory findings were remarkable for hypocalcemia and elevated alkaline phosphatase (Table). Radiographs of her extremities revealed multiple healed and healing fractures (Image 1), initially raising concern for non-accidental trauma. Further review of films with radiology revealed generalized bony demineralization, widened growth plates and metaphyseal fraying and flaring consistent with the diagnosis of rickets (Image 2). No vertebral compression fractures were noted, nor was rachitic rosary noted on chest radiographs. An elevated intact parathyroid hormone level (PTH) and extremely low serum 25-hydroxyvitamin D (25-OH vitamin D) concentration (Table) confirmed a diagnosis of severe hypocalcemic rickets due to vitamin D deficiency.

Endocrinology was consulted and elicited on history that she was a picky eater, only eating rice, fries and potato chips. She drank homemade green juices and smoothies but had limited dairy intake. On further review of systems, the family had not noticed any muscle spasms, seizures or twitching. Physical exam by the endocrinologist revealed widened wrists and ankles and rachitic rosary-prominent costochondral junctions of the ribs. She had no symptoms of neuromuscular irritability (negative Chvostek sign) despite ionized calcium of only 0.93 millimoles/L, highlighting the chronicity of the presentation.

**CPC-EM Capsule**

What do we already know about this clinical entity?

Nutritional rickets commonly presents at an early age with delayed closure of the fontanelle, frontoparietal bossing of the skull, bowing of the legs, widening of the wrists, and craniotabes.

What makes this presentation of disease reportable?

Late presentation without overt and classical signs and symptoms. The initial differential focused on non-accidental trauma (NAT) and did not entertain rickets.

What is the major learning point?

While NAT should remain high on the differential diagnosis, it’s important to remain mindful of the increased risk of nutritional rickets in patients with autism and developmental delay.

How might this improve emergency medicine practice?

With early diagnosis and management in the Emergency Department, Vitamin D deficiency causing rickets can be easily treated and serious complications avoided.

The patient received calcium carbonate and calcitriol therapy followed by gradual supplementation of vitamin D3. She experienced significant improvement of pain and gait issues one month after initiation of calcitriol, calcium carbonate, and vitamin D3 supplementation along with orthotics. She was followed by endocrinology and was taken off calcitriol with normalization of calcium and serum 25-OH vitamin D concentrations in a month. One year after the diagnosis, calcium supplementation was also stopped with complete normalization of her calcium (10.1 mg/dL), PTH (38.6 picogram/mL), alkaline phosphatase (355 units/L) and vitamin D (48.7 nanogram/mL). All fractures were well healed except for a malaligned right humerus with no functional disability. She had improved dietary calcium intake with supplemental nutritional shakes and remained on 2,000 IU of vitamin D3 daily.

## DISCUSSION

Rickets is a metabolic bone disease marked by failure of bone mineralization in the growth plates. The disease had been largely eradicated in the United States since the 1930s, with a hospitalization rate of 1.23 per 100,000 in children younger than 10 years old.^1^ Another study showed the prevalence of nutritional rickets to be approximately nine cases per one million children.^2^ The Centers for Disease Control and Prevention reports a rate of five cases per one million children aged six months to five years.^3^ Causes of calcipenic rickets include nutritional deficiency of calcium and vitamin D (the most common cause) and genetic abnormalities in the enzyme 25(OH)D3-1-alpha-hydroxylase or vitamin D receptors. Other causes of rickets include malabsorption syndromes, medications, hypophosphatemic rickets, or renal disease.

Common presenting clinical signs of calcipenic rickets include bowing of the legs, frontal bossing, generalized hypotonia, Harrison’s groove formation, pigeon-breast deformity, and rachitic rosary.^4^ Defects in bony mineralization result in an increase in osteoid and therefore an un-mineralized organic bone matrix that loses stability and strength.^5^ At the growth plate, chondrocytes become hypertrophic and disorganized with thickening of the growth plate and hypertrophic zone expansion.^6^ Typical age at presentation is in toddlers with the characteristic bowed legs when they start weight bearing.

Our patient’s presentation was more insidious. She did not present with the classic physical exam findings. Instead she had chronic extremity pain at nine years of age. Generally, bone pain is the first symptom on presentation in pediatric patients with acute lymphoblastic leukemia.^7^ Bone pain was also the initial complaint in 67.5% of patients with primary lymphoma.^8^ When children present with chronic bone pain to the ED, clinical suspicion is generally high for physical and sexual abuse, musculoskeletal abnormalities, hematologic processes, or malignancy.^15^ Children with disabilities are at higher risk for physical/emotional abuse than those without disabilities.^9^,^10^ Hence, we entertained this diagnosis in the pediatric ED while the workup was ongoing and the Child Protective Team was consulted.

With its decreasing incidence in the developed world, nutritional rickets due to vitamin D deficiency was not high on the initial differential. However, children with autism must be evaluated on different premises. In fact, children with autism and developmental delay are five times more likely to have feeding difficulty, or specific feeding behaviors, and are particularly susceptible to calcium deficiencies.^11^ Food selection in children with autism is often based on color and texture preferences, in addition to aversions to dairy products or foods rich in protein. Deficiencies have been observed in vitamins A, B6, B12, C, D, and K.^12^ It has also been observed that children with autism have significantly lower vitamin D levels compared to those without autism and that children with vitamin D-dependent rickets have autistic markers, which have resolved after supplementation with vitamin D.^13^

## CONCLUSION

Although the differential diagnosis of a child presenting with bone pain includes an extensive list of conditions ranging from benign growing pains to malignancies, in children with developmental delay, failure to thrive, or other musculoskeletal abnormalities, non-accidental trauma, and nutritional rickets should be high on the differential diagnosis. It is important for physicians to remain mindful of the increased risk of nutritional rickets in patients with autism and developmental delay. Vitamin D deficiency causing rickets can be easily treated and serious complications avoided with early diagnosis and management.

Documented patient informed consent and/or Institutional Review Board approval has been obtained and filed for publication of this case report.

## Figures and Tables

**Table:** Initial laboratory data for nine-year-old patient presenting with chronic episodic bone pain.

Laboratory test	Value	Reference range
Serum calcium (mg/dL)	6.4	9.1–10.5
Alkaline phosphatase (IU/L)	1847	134–349
Intact parathyroid hormone (PTH) (pg/mL)	1521.3	8.7–77.1
25-hydroxy vitamin D (ng/mL)	2.8	30–100
Ionized calcium (mmol/L)	0.93	2.2–2.7
Phosphorous (mg/dL)	3.6	2.5–4.5
Albumin (g/dL)	4.5	3.5–5.5

*mg*, milligram; *dL,* deci-liter; *IU,* international units; *pg,* picogram; *mL,* milliliter; *mmol,* millimoles; *L,* liter; *g,* gram.
